# A Small RNA That Neutralizes a Protein Linked to Tumor Development

**DOI:** 10.1371/journal.pbio.0030147

**Published:** 2005-03-22

For most of human history, cancer has been incurable. But with the invention of anesthesia in the mid-19th century, surgeons were able to remove some forms of cancer surgically. Radiotherapy arrived next, soon after the discovery of X rays in 1896. Chemotherapy, now a mainstay of cancer treatment, did not arrive until the mid-1940s, when nitrogen mustard, an alkylating agent related to the mustard gas used in the two World Wars, was developed as an anticancer agent. Unfortunately, although cancer cells are hypersensitive to the effects of alkylating agents—molecules that introduce lethal changes into the cell's DNA—normal cells are also targeted by them, although less damage is caused because normal cells typically divide slower than cancer cells. Using chemotherapy based on alkylating agents to treat cancer is like using a sledgehammer to crack a nut. But with improved knowledge about how cancer cells differ from normal cells, chemotherapeutics are now being designed that hit only cancer cells.

Many of these new chemotherapeutics target protein receptors called tyrosine kinases. These receptors, which sit on the cell surface, normally stimulate intracellular pathways that control proliferation and other cellular functions in response to growth factors. In tumors, these receptors often have mutations that allow them to become active without growth factor binding, which results in the uncontrolled proliferation that is characteristic of cancer cells. For instance, mutations in the RET receptor tyrosine kinase are responsible for multiple endocrine neoplasia (MEN) type 2 syndromes. Whereas external stimulation by a growth factor is normally needed before two RET molecules can bind together (a process called dimerization) to activate intracellular signaling cascades, in MEN type 2A, a mutation in the RET receptor tyrosine kinase provokes (or induces) dimerization without external stimulation.

In recent years, several proteins and various small synthetic chemicals have been designed that specifically inhibit the activity of mutated receptor tyrosine kinases and show anticancer activity. Domenico Libri and colleagues are now working on another class of molecules, called aptamers, that have potential as anticancer drugs. Aptamers—single-stranded nucleic acid molecules that are 50–100 bases long and can be selected for their ability to bind directly and tightly to specific proteins—are less likely to be targeted and destroyed by the body's natural defenses than some other types of potential therapeutic molecules.

To find an aptamer able to recognize the RET receptor kinase within a cellular membrane environment, the researchers used whole-cell SELEX (systematic evolution of ligands by exponential enrichment), a process in which large pools of oligonucleotides are enriched for molecules that can distinguish between a real and sham target. First, they incubated a large pool of RNAs with PC12 cells, a rat cell line not expressing RET, to remove sequences binding non-specifically to the PC12 cell surface. Unbound sequences were recovered and applied to PC12 cells expressing human RET with the MEN type 2A mutation that causes dimerization. This time, bound sequences were retained, and the whole selection process was repeated another 14 times to select for aptamers that recognize the dimeric form of the RET extracellular domain.[Fig pbio-0030147-g001]

**Figure pbio-0030147-g001:**
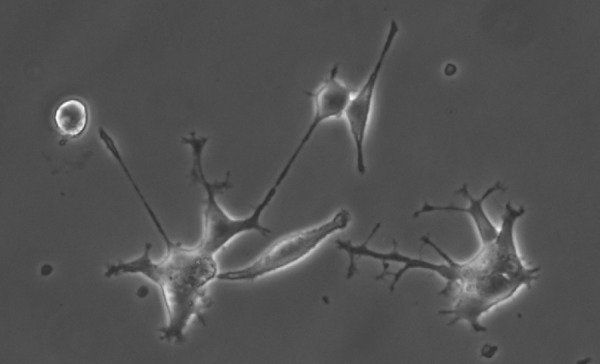
A newly synthesized molecule, D4, inhibits cellular differentiation

Of the 67 sequences pulled out of the final pool of RNAs, the researchers found one sequence, D4, that not only bound the extracellular domain of RET but also blocked RET downstream signaling events and subsequent cellular and molecular changes. The researchers suggest that D4 blocks the dimerization-dependent activation of RET—whether it's induced by its physiological signaling molecule or by an activating mutation—and suggest that their method can be used to identify macromolecules with potential therapeutic effects against other transmembrane receptors involved in tumorigenesis, particularly since the whole-cell SELEX approach should efficiently select aptamers that recognize these receptors as they are found on the surface of tumor cells.

